# Diabetes mellitus with anti-glomerular basement membrane disease and anti-tubular basement membrane disease: a rare case report of reversible dialysis dependence

**DOI:** 10.3389/fimmu.2025.1691810

**Published:** 2026-01-12

**Authors:** Junqi Hu, Yuanzi Liang, Kebing Sun, Yuefei Xiao

**Affiliations:** 1 Department of Nephrology, Aerospace Center Hospital, Beijing, China; 2 Department of Radiology, Aerospace Center Hospital, Beijing, China

**Keywords:** anti-glomerular basement membrane (GBM) disease, anti-tubular basement membrane (TBM) disease, diabetic nephropathy, dialysis (ESRD), renal biopsy

## Abstract

We present an instructive case of an elderly woman with a history of diabetes and diabetic retinopathy who was admitted due to rapidly progressive renal failure requiring dialysis. Although diabetic nephropathy was initially suspected, the patient’s rapid clinical decline, significant hematuria, and proteinuria inconsistent with typical diabetic nephropathy prompted further serological evaluation and renal biopsy. Pathological examination revealed the rare coexistence of anti-glomerular basement membrane disease and anti-tubular basement membrane-associated interstitial nephritis. Following aggressive immunosuppressive therapy and plasma exchange, the patient was successfully weaned off dialysis with marked improvement in renal function. This case highlights the importance of comprehensive assessment in diabetic patients with atypical renal presentations and underscores the critical role of renal biopsy in guiding treatment decisions.

## Introduction

Diabetic nephropathy is the leading cause of end-stage renal disease worldwide. While renal histology serves as the gold standard for diagnosis, its invasive nature limits clinical application, making diagnosis largely dependent on clinical history and laboratory indicators. Diabetic nephropathy was once thought to follow a classic “proteinuria-first” progression, but this model was primarily based on observations in type 1 diabetes. Today, especially in type 2 diabetes with higher prevalence, the clinical presentation of this disease exhibits significant heterogeneity (1). Studies indicate that up to 40% of patients present with progressive renal decline without significant proteinuria, termed “non-proteinuric diabetic nephropathy.” This makes relying solely on proteinuria as a screening indicator prone to significant underdiagnosis. Another crucial diagnostic clue is diabetic retinopathy. While its presence strongly suggests diabetic kidney damage, the two conditions do not necessarily coexist; the absence of retinopathy does not rule out renal involvement (2). Thus, diabetic nephropathy represents a complex spectrum of disease. Diagnosis requires comprehensive assessment of renal function and proteinuria, coupled with vigilance for atypical cases to avoid misdiagnosis and missed diagnosis.

## Case report

A 68-year-old female was admitted due to shortness of breath and loss of appetite for over one month, along with elevated serum creatinine levels for 10 days. One month prior, she developed dyspnea without obvious cause, accompanied by significant anorexia, intermittent nausea and vomiting, subjectively decreased urine output, and bilateral lower extremity edema. No gross hematuria or urinary symptoms such as frequency, urgency, or dysuria were reported. Ten days before admission, local laboratory tests revealed serum creatinine ≈600 μmol/L, hyperkalemia, and anemia. Referral to a tertiary hospital suggested chronic kidney insufficiency of uncertain etiology. Four days pre-admission, serum creatinine reached ≈863 μmol/L, urine output was ≈250 ml/day, and dialysis was recommended. She was admitted to our department for dialysis initiation. Since symptom onset, she denied fever, cough, sputum, hemoptysis, dizziness, headache, blurred vision, photosensitivity, alopecia, oral ulcers, or joint pain. Mental status, appetite, and sleep were poor; bowel habits were normal. Recent weight was stable. Past medical history included type 2 diabetes for 8 years, diabetic retinopathy with bilateral blindness, metformin and glipizide use for glycemic control, left eye cataract surgery, glaucoma, diclofenac use for periarthritis, chronic poor sleep managed with eszopiclone, no history of infectious or other chronic diseases, cesarean section, no trauma, and no blood product transfusions.

Physical examination on admission: Temperature 36.4 °C, pulse 86 bpm, respiratory rate 20 bpm, blood pressure 141/83 mmHg. Conscious but fatigued; no pharyngeal erythema or swelling; clear lung sounds; no dry or wet rales; no pleural friction rub. Heart rate 86 bpm, rhythm regular, no pathological murmurs. Abdomen soft, non-tender, no rebound tenderness. Liver and spleen not palpable; no renal percussion tenderness; normal bowel sounds. Moderate pitting edema in both lower limbs.

Urinary system ultrasound: Right kidney measures 9.8 × 5.0 × 5.8 cm, with a parenchymal thickness of 1.5 cm. Left kidney measures 10.2 × 4.9 × 5.3 cm, with a parenchymal thickness of 1.4 cm. Renal vascular ultrasound: Blood flow in both renal arteries and veins is unobstructed. Cardiac ultrasound: Left ventricular ejection fraction (EF) 57%, no significant abnormalities in cardiac structure or blood flow. Pulmonary CT: Bilateral pleural effusion with incomplete expansion of adjacent lung tissue, no signs of pulmonary hemorrhage were observed. Laboratory test results are shown in (**Table 1**).

**Table 1:** Relevant assistant examinations upon admission.

	Parameter	Result
Biochemistry	Cr	1207.6 μmol/L
	BUN	42.5 mmol/L
	K	5.22 mmol/L
	Ca	2.09 mmol/L
	P	2.61 mmol/L
	Alb	29.7 g/L
	ALT	0 U/L
	AST	7.6 U/L
	TB	14.1 μmol/L
	DB	4.4 μmol/L
	IDB	9.7 μmol/L
	LDH	155 U/L
Urinalysis	SG	1.009
	PRO	1+
	BLD	3+
	RBC	210/HP
	WBC	8.55/HP
	Urine osmolality	314mOsm/kg.water
Early Kidney Injury Markers	Urinary microalbumin	134.53 mg/L
	Urinary transferrin	5.59 mg/L
	Urinary immunoglobulin G	23.27 mg/L
	Urinary α1-microglobulin	10.51 mg/L
	Urease (NAG)	10.75 IU/L
Related Immunology	Anti-GBM	84.0 cu
	Anti-PLA2R	negative
	Immunoglobulin	normal
	Complement	normal
	Serum light chain combination	normal
	Serum immunofixation electrophoresis	negative
	urine immunofixation electrophoresis	negative
	ANCA	negative
	Antinuclear antibody	negative
Complete blood count	WBC	4.95 × 10^9/L
	NE%	78.3%
	Hb	72 g/L
	PLT	147 × 10^9/L
	CRP	74.62 mg/L
Cardiac markers	BNP	434 pg/mL
	CK-MB	1.72 ng/mL
	cTnI	0.012 pg/mL
Lymphocyte subsets	CD3	60.4%
	CD4	32.5%
	CD8	25.6%
	CD4/CD8 ratio	1.27
	B lymphocyte percentage	30.9%
	NK cell percentage	9.1%
	Total T lymphocyte count	153/uL
	Helper T lymphocyte count	82/uL
	Suppressor T lymphocyte count	65/uL
	B lymphocyte count	79/uL
	NK cell count	23/uL
Other	Glycated hemoglobin	7.6%
	DD	2443 μg/L
	PTH	251.44 pg/mL

**Figure 1:**
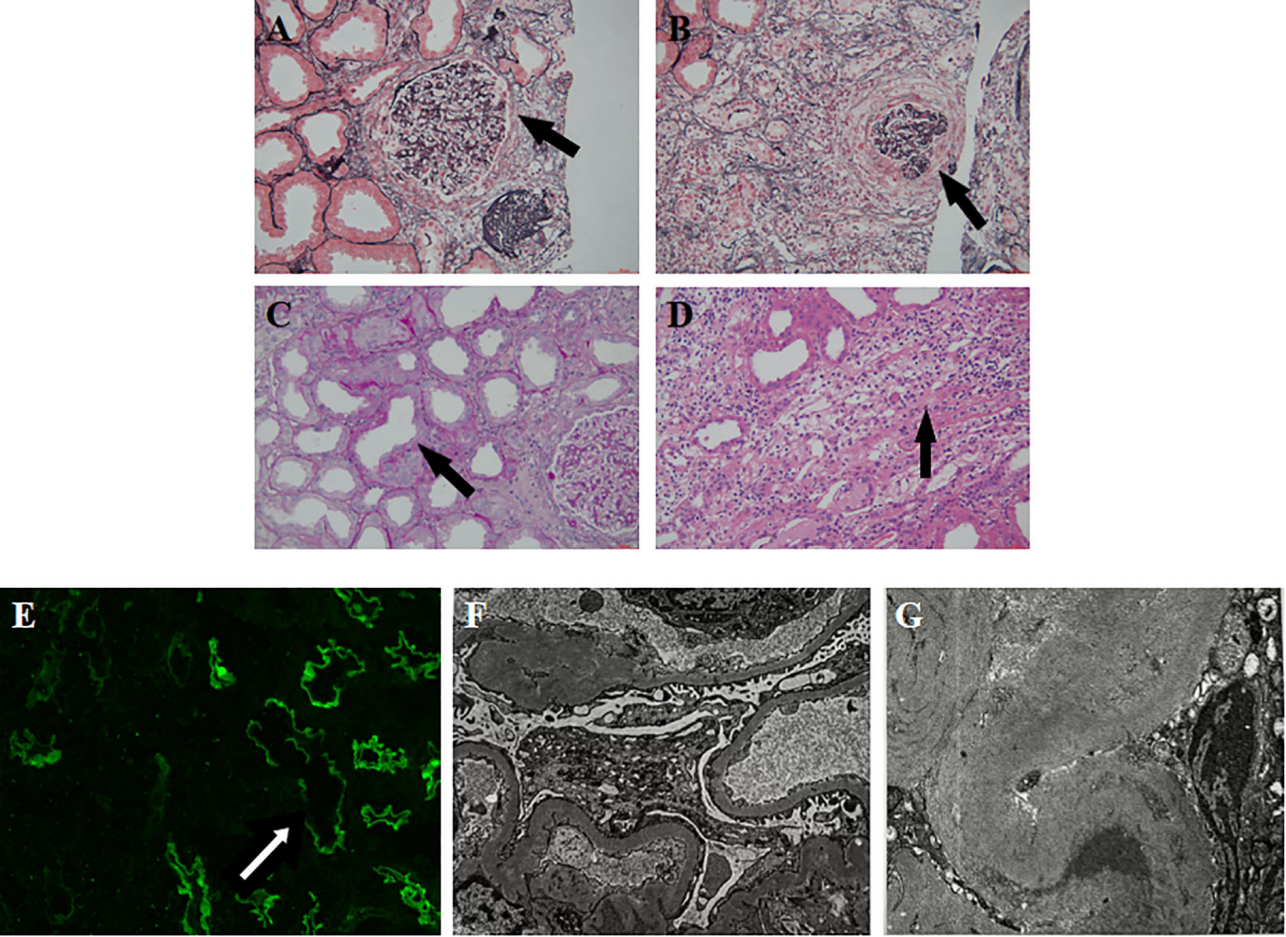
**(A)** Partial destruction of the Bowman’s capsule in the glomerulus. **(B)** Cellular crescent formation. **(C)** Tubular epithelial vacuolization, granular degeneration, brush border loss, atrophy, and protein casts. **(D)** Interstitial lymphocytic, mononuclear, and neutrophilic infiltration with fibrosis and tubulitis. **(E)** Paraffin fluorescence: No distinct linear or granular IgG deposits were observed in the glomerular capillary walls, segmental TBM linear deposits +. **(F, G)** Electron microscopy: mesangial hyperplasia, basement membrane changes, tubular degeneration, and interstitial inflammation.

Renal pathology (**Figure 1**): Light microscopy findings: Renal biopsy specimens revealed 14 glomeruli, with 2 showing ischemic sclerosis. The remaining glomeruli exhibited mild segmental proliferation of mesangial cells and matrix, vacuolar degeneration of the basement membrane, formation of 2 cellular crescents, partial destruction of Bowman’s capsule, and ischemic atrophy in some glomeruli. Renal tubular epithelial cells show vacuolar and granular degeneration, focal loss of brush border, and multifocal atrophy. A few protein casts are present in the lumen. The renal interstitium shows multifocal infiltration of lymphocytes, mononuclear cells, and some neutrophils, accompanied by fibrosis, with tubulitis formation. Small arterial walls are thickened with fibrous proliferation and sclerosis of the intima. Congo red staining is negative. Immunohistochemistry: C4d++-+++, with granular and linear deposits on capillary walls and in some TBM. CD3 focal ++, CD20 focal ++, CD68 focal ++, CD138 negative. IgG1 negative, IgG2 negative, IgG3 negative, IgG4 negative. Paraffin fluorescence: No distinct linear or granular IgG deposits were observed in the glomerular capillary walls, segmental TBM linear deposits +.Electron microscopy: Mild segmental hyperplasia of glomerular mesangial cells and matrix, minimal electron-dense deposits within the basement membrane, homogeneous thickening of small segments of the basement membrane, and segmental fusion of epithelial foot processes. Renal tubular epithelial cells show vacuolar degeneration with increased lysosomes, partial loss of microvilli, partial atrophy, and protein casts visible in some tubular lumens. The basal membranes of segmental renal tubules show minimal electron-dense deposits. The renal interstitium shows significant lymphocytic and mononuclear cell infiltration with collagen fiber proliferation.

Pathological diagnosis: 1. Focal proliferative glomerulonephritis with partial crescent formation, not excluding anti-GBM disease; 2. Acute tubulointerstitial nephritis, with a high likelihood of anti-TBM-associated interstitial nephritis; 3. Ischemic kidney injury.

Treatment history: The patient’s renal function deteriorated progressively, and hemodialysis therapy was initiated. Based on the renal pathology results, comprehensive treatment including plasma exchange therapy and steroid pulse therapy was administered. 3 to 16 days after admission: A total of six steroid pulse therapies with 500 mg of methylprednisolone were administered; Plasma exchange therapy was administered six times, with a total of 12,300 ml of plasma exchanged. On the eighth day after admission, the patient was prescribed oral prednisone 50 mg daily. On the 13th day after admission, serum creatinine levels fluctuated around 200 μmol/L, urine output gradually returned to normal, and the frequency of dialysis was gradually reduced. No further dialysis was required starting on the 52nd day after admission. On the 58th day after admission, the anti-GBM antibody test was negative. Follow-up: The patient was followed up at our outpatient clinic three months and six months after discharge. Laboratory tests showed serum creatinine levels of 213 μmol/L and 202 μmol/L, respectively, with adequate urine output, and no further dialysis was required.

## Discussion

Anti-glomerular basement membrane (GBM) disease is a type of small vessel vasculitis, where circulating antibodies target and attack the intrinsic antigens of the GBM and alveolar basement membrane (ABM), leading to rapidly progressive glomerulonephritis and/or alveolar hemorrhage. Anti-GBM disease is rare, with an estimated incidence of less than 2 per million (3). Large biopsy studies have shown that approximately 15% of all cases of crescentic glomerulonephritis are caused by anti-GBM disease (4). However, anti-GBM disease rarely leads to ESRD overall, it frequently results in ESRD in affected individuals, particularly with delayed diagnosis or treatment. Under light microscopy, crescentic glomerulonephritis is typically observed, while immunofluorescence microscopy reveals nearly diagnostic findings, namely linear IgG deposits along the glomerular capillaries (occasionally along the distal tubules).Anti-tubular basement membrane (TBM) antibody disease is relatively rare and can manifest as acute or chronic kidney injury. Immunofluorescence of the tubular basement membrane (TBM) shows strong diffuse linear staining with IgG, and variable staining with C3 (5–8). Anti-tubular basement membrane (TBM) disease is a condition characterized by selective deposition of immunoglobulins on the tubular basement membrane, associated with autoantibodies targeting an antigen known as tubulointerstitial nephritis antigen, which is expressed exclusively in the kidneys. Two cases of anti-TBM antibody nephritis have been reported in the literature. Andres et al. (9) and Brentjens et al. (10) reported that in anti-TBM antibody nephritis, significant tubulointerstitial nephritis can be observed, with linear immunofluorescent staining of immunoglobulins along the TBM visible under immunofluorescence microscopy. Anti-TBM antibodies may be present with or without anti-glomerular basement membrane antibodies (5, 6). Anti-tubular basement membrane antibody disease may occur without a specific cause (11), but it can also be detected in other kidney diseases, such as systemic lupus erythematosus nephritis (12), Sjögren’s syndrome (13), kidney transplant rejection (14, 15), post-streptococcal glomerulonephritis (16), and drug-induced tubulointerstitial nephritis caused by methicillin (17). It is worth noting that some patients with membranous nephropathy may also have evidence of anti-TBM antibodies (8), with males being more susceptible than females, and most patients developing the condition before the age of 5 (18).

Diabetic patients are at high risk for chronic kidney disease, but studies indicate that a minority of renal lesions in this population are non-diabetic kidney disease. Renal biopsies are rarely performed in patients with diabetes and CKD, so diabetic kidney disease is typically a clinical (presumptive) diagnosis. Consequently, when clinical presentations are atypical—such as rapidly progressive renal deterioration (over weeks to months), active urine sediment (particularly marked microscopic hematuria or red blood cell casts), proteinuria severity inconsistent with diabetes duration, and acute kidney injury without apparent cause—these serve as critical warning signs. In such cases, actively pursuing etiology is paramount. In many regions, the medical consensus is to avoid kidney biopsies in diabetic patients, but this approach may also lead to undiagnosed cases of non-diabetic kidney disease (19–21).Approximately one-quarter (620 cases) of patients in a single-center study cohort had diabetes (22). Among these patients, one-third each presented with isolated typical diabetic glomerular disease, diabetic glomerular disease combined with non-diabetic kidney disease, and isolated non-diabetic kidney disease. The most common non-diabetic kidney diseases were acute tubular necrosis (28%), immune-mediated glomerular disease (25%), hypertensive nephrosclerosis (18%), and focal segmental glomerulosclerosis (18%).

The patient’s original hospital had diagnosed diabetic nephropathy and planned to initiate maintenance hemodialysis treatment. However, the appropriateness of diagnosing diabetic nephropathy based solely on clinical manifestations and initiating comprehensive chronic kidney disease treatment was questionable. We therefore decided to create conditions to perform a renal biopsy for pathological examination. The pathological report considered anti-GBM disease not excluded and anti-TBM-related interstitial nephritis possible. Once anti-GBM disease and anti-TBM disease is considered for diagnosis, plasma exchange combined with immunosuppression is recommended. Plasma exchange removes circulating anti-GBM antibodies and other inflammatory mediators, while immunosuppressive agents minimize the formation of new antibodies. Overall, existing data indicate that 30%–45% of patients receiving plasmapheresis plus immunosuppression will not progress to end-stage renal disease (ESRD) or death (23–27). However, patients initiating treatment before developing oliguria or anuria show significantly greater likelihood of recovery, whereas those requiring dialysis or exhibiting crescent formation in all glomeruli rarely recover. Notably, the immunofluorescence renal biopsy in this patient did not reveal the typical continuous linear IgG deposition along the glomerular capillary walls characteristic of anti-GBM disease. This may be related to antibody titer or a rare antigenic epitope specificity. However, the combination of strongly positive serum anti-GBM antibodies (84.0 cu), visible cellular crescents under light microscopy, and a dramatic clinical response to immunosuppressive therapy and plasma exchange collectively supports the diagnosis of anti-GBM disease. This phenomenon underscores the necessity of integrating serology, histopathology, and clinical response to treatment for a comprehensive diagnosis of such conditions, rather than relying solely on a single pathological indicator. We actively administered hormone and plasma exchange therapy, and the patient’s renal function gradually improved. The patient ultimately ceased dialysis, achieving significant benefits in terms of both quality of life and economic outcomes. The most important lesson from this case is that when diabetic nephropathy patients have conditions that allow it or exhibit clinical manifestations inconsistent with diabetic nephropathy, every effort should be made to perform kidney biopsy pathology examinations, which may save a pair of kidneys and a family.

## Data Availability

The raw data supporting the conclusions of this article will be made available by the authors, without undue reservation.
